# Hemodynamic Changes during a Deep Inspiration Maneuver Predict Fluid Responsiveness in Spontaneously Breathing Patients

**DOI:** 10.1155/2012/191807

**Published:** 2011-12-13

**Authors:** Sébastien Préau, Florent Dewavrin, Vincent Soland, Perrine Bortolotti, Delphine Colling, Jean-luc Chagnon, Alain Durocher, Fabienne Saulnier

**Affiliations:** ^1^Service de Réanimation Polyvalente, Centre Hospitalier Jean Bernard, Avenue Désandrouin, 59300 Valenciennes, France; ^2^Service de Réanimation Médicale et Médecine Hyperbare, Hopital Albert Calmette, CHRU de Lille, Boulevard du Professeur J. Leclercq, 59037 Lille Cedex, France; ^3^Université Lille Nord de France, EA 3614, 59000 Lille, France

## Abstract

*Objective*. We hypothesized that the hemodynamic response to a deep inspiration maneuver (DIM) indicates fluid responsiveness in spontaneously breathing (SB) patients. *Design*. Prospective study. *Setting*. ICU of a general hospital. *Patients*. Consecutive nonintubated patients without mechanical ventilation, considered for volume expansion (VE). *Intervention*. We assessed hemodynamic status at baseline and after VE. *Measurements and Main Results*. We measured radial pulse pressure (PP) using an arterial catheter and peak velocity of femoral artery flow (VF) using continuous Doppler. Changes in PP and VF induced by a DIM (ΔPPdim and ΔVFdim) were calculated in 23 patients. ΔPPdim and ΔVFdim ≥12% predicted responders to VE with sensitivity of 90% and specificity of 100%. *Conclusions*. In a restricted population of SB patients with severe sepsis or acute pancreatitis, ΔPPdim and ΔVFdim are accurate indices for predicting fluid responsiveness. These results should be confirmed in a larger population before validating their use in current practice.

## 1. Introduction

 Blood volume is a determinant of hemodynamic stability, which regulates oxygen supply to the tissues. Volume expansion (VE) is frequently the first-line therapeutic measure for improving the hemodynamic status of patients with acute circulatory failure. Absence of  VE and excessive fluid loading can lead to inadequate tissue oxygenation, organ failure, and sometimes death [1]. Unfortunately, only 40–70% of critically ill patients with acute circulatory failure significantly increase their stroke volume (SV) in response to VE regardless of the respiratory conditions [2]. This emphasizes the need for factors that predict fluid responsiveness in order to distinguish patients who might benefit from VE, as well as to avoid ineffective VE.

Cardiac preload estimation is not accurate for predicting fluid responsiveness in patients with acute circulatory failure [3]. Dynamic indices, based on the analysis of SV preload dependence, have been validated to predict fluid responsiveness in mechanically ventilated patients [3]. However, only a few studies, yielding conflicting results, tested VE responsiveness indices in spontaneously breathing (SB) patients [4–8].

The passive leg-raising maneuver has been reported to provide valid assessment of fluid responsiveness in a broad population, including patients with cardiac arrhythmias or spontaneous respiratory movements [9]. Nevertheless, depending on the method used, this test may not increase cardiac preload enough to detect preload dependence and/or may not be possible to perform with all types of beds and stretchers [9, 10].

During spontaneous breathing (SB), inspiration decreases intrathoracic pressure and increases intra-abdominal pressure, increasing the preload of the right ventricle, resulting in an increase in right ventricular SV, and an expiratory increase in left ventricular SV [11–13] if the heart is preload-responsive. As reported during mechanical ventilation using low tidal volume, possibly masking biventricular preload dependence [14–16], respiratory changes in intrathoracic pressure during SB may be insufficient to modify loading conditions of the ventricles to the extent that respiratory changes in left ventricular SV can be measured [4]. Consequently, a deep inspiration maneuver (DIM) might improve the predictive value of SB-induced SV variations for detecting fluid responsiveness. To our knowledge, DIM-induced hemodynamic changes have never been previously tested for detecting fluid responsiveness in SB patients.

As previously described, the velocity peak of femoral artery flow (VF) and radial pulse pressure (PP) are reliable surrogates of left ventricular SV for detecting SV changes during preload responsiveness assessment [7].

We thus conducted a prospective study to assess whether DIM-induced changes in PP and VF (ΔPPdim and ΔVFdim, resp.) can predict fluid responsiveness in SB patients with acute circulatory failure. Moreover, to determine physiological hemodynamic changes during the specific DIM used in this study, ΔPPdim and ΔVFdim were assessed in 6 healthy volunteers prior to patient analyses. 

## 2. Patients and Methods

### 2.1. Ethical Considerations

This study was submitted to the institutional review board for human subjects of our institutions. Protocol was approved and considered to be part of the routine practice. Healthy subjects and patients gave informed consent prior to inclusion in the study. Healthy subjects and consecutive patients hospitalized in the intensive care unit of the General Hospital Center in Valenciennes (France) were prospectively assessed for a 12-month period until February 2009.

### 2.2. Healthy Volunteers

Criteria for inclusion of healthy volunteers included no chronic diseases and a stable physical state for at least 6 weeks prior to the study. Subjects were examined after overnight fasting.

### 2.3. Patients

We selected for inclusion all nonintubated SB patients without ventilatory support and with acute circulatory failure, for whom the attending physician decided to perform fluid challenge. This decision was based on the presence of at least one clinical sign of inadequate tissue perfusion and absence of contraindications for fluid infusion. Clinical signs of inadequate tissue perfusion were defined as follows: systolic arterial pressure (SAP) of 90 mm Hg (or a decrease of 40 mm Hg in previously hypertensive patients), urine output of 0.5 mL/kg/h for at least 1 h, tachycardia (heart rate ≥100/min), and mottled skin. Cardiac rhythm had to be regular. Each patient had a 3-Fr radial catheter (Seldiflex Plastimed; Division Prodimed, Saint-Leu-La-Foret, France) inserted prior to the study as part of standard hemodynamic monitoring.

Patients were not included in the study if they displayed accessory muscle use (sternocleidomastoid, scalene, pectoralis major, trapezius, internal intercostals, and abdominal muscles), if the respiratory rate was over 30 or if they could not sustain an inspiration strain for over 5 s.

Eligible patients were secondarily excluded if they had high-grade aortic insufficiency, if transthoracic echogenicity was not satisfactory, or if mechanical ventilation was warranted.

### 2.4. Measurements (Systemic Arterial Pressure, Stroke Volume, Femoral Artery Flow)

Noninvasive (healthy volunteers) and invasive (patients) arterial pressures, heart rate, and respiratory rate were measured with offline recordings on a central monitor (Information Center M3155; Philips Medical System, Andover, MA, USA) connected to bedside monitors (IntelliVue MP70; Philips Medical System, Boeblingen, Germany). For respiratory rate measurements, thoracic impedance recordings were used.

For patients, systolic and diastolic arterial pressures (SAP and DAP) were measured with a radial catheter. Mean arterial pressure (MAP) was calculated as MAP = (SAP + 2DAP)/3. Arterial PP was calculated as SAP minus DAP.

All echographic measurements were made on-line with commercially available echocardiographic HDI 3000 equipment (Philips Medical System; Bothell, WA, USA) with a 2-MHz transthoracic transducer. Aortic blood flow was recorded with a pulsed Doppler at the aortic valve so that the click of aortic closure was obtained. The velocity time integral of aortic blood flow was measured. The aortic valve area was calculated from the diameter of the aortic orifice, measured at insertion of the aortic cusps, as aortic area = π* aortic diameter²/4. SV was calculated as SV = aortic valve area * the velocity time integral of aortic blood flow [18].

Femoral blood flow was recorded with a continuous Doppler at the common femoral artery. One of the two common femoral arteries was identified with echographic 2-dimensional and color Doppler's modes. VF was measured with a continuous Doppler.

An average of 10 consecutive cardiac cycles over at least one respiratory cycle was used for measurement of SAP, DAP, MAP, PP, SV, and VF.

### 2.5. Respiratory Variations during Quiet SB

Maximal and minimal values for PP and VF were determined over a respiratory cycle during quiet SB. Respiratory variations in PP and VF (ΔPP and ΔVF, resp.) were calculated as previously described [19]: respiratory variation within a respiratory cycle = (maximal value − minimal value)/((maximal value − minimal value)/2). Three consecutive measurements were averaged.

### 2.6. Respiratory Variations during DIM

All patients received a brief training (<5 min) to make them familiar with the performance of DIM. After passive exhalation, DIM consisted of slow continuous inspiration strain (5–8 s) followed by slow passive exhalation. Then, normal quiet breathing was resumed. Inspiration and exhalation durations were controlled at the bedside with the echograph chronometer and off-line with thoracic impedance recording. Maximal values of DIM-induced PP and VF were recorded as the maximal value of PP and VF during the deep inspiration strain and the following exhalation. DIM-induced changes in PP and VF (ΔPPdim and ΔVFdim, resp.) were calculated as follow.

DIM-induced changes = (maximal value during DIM − minimal value during quiet SB prior to DIM)/((maximal value during DIM − minimal value during quiet SB prior to DIM)/2). Three consecutive measurements were averaged. The variability of ΔPPdim and ΔVFdim measurements was tested. ΔVFdim was measured three times in all healthy volunteers by the same observer (intraobserver variability) and by a second observer (interobserver variability). ΔPPdim and ΔVFdim were measured three times in 10 patients by the same observer (intraobserver variability).

### 2.7. Study Design

Patients were studied in a semirecumbent position. Supportive therapies and vasopressors, if present, remained unchanged throughout the study. All hemodynamic and echocardiographic measurements during quiet SB and DIM were performed at baseline and immediately after a 30 min VE using 500 mL of 6% hydroxyethyl starch. Patients were considered as responders to VE if their SV increased by 15%. Because the aortic valve area is not affected by VE, this 15% cut-off value was defined prior to beginning the study as twice the intraobserver variability of the velocity time integral of aortic valve flow, measured by transthoracic echocardiography in previous studies [4–7]. Tested parameters and SV were recorded consecutively within 5 min by 2 different investigators, before and after VE.

### 2.8. Statistical Analysis

Numerical data are given as means ± SD unless otherwise indicated. The Shapiro-Wilk test was used to test for normal distribution. Comparison of means within groups was performed using a paired-sample Student's *t-*test or a paired-sample Wilcoxon's test. Comparison of means between groups was performed using an independent sample Student's *t-*test or Mann-Whitney's *U-*test. Qualitative variables were reported as number and percentage and compared between groups using a Fisher test. Linear correlations were tested using the Pearson test. Receiver-operating characteristic curves ± SE were compared using the Hanley-McNeil test [20]. Cut-off values for ΔPP, ΔPPdim, ΔVF, and ΔVFdim were chosen to correspond to the best respective Youden's index [21]. A *P* ≤ .05 was considered statistically significant. Statistical analysis was performed using SPSS 13.0.1 software (SPSS, Chicago, IL, USA).

## 3. Results

### 3.1. Healthy Volunteers

**Figure 1:**
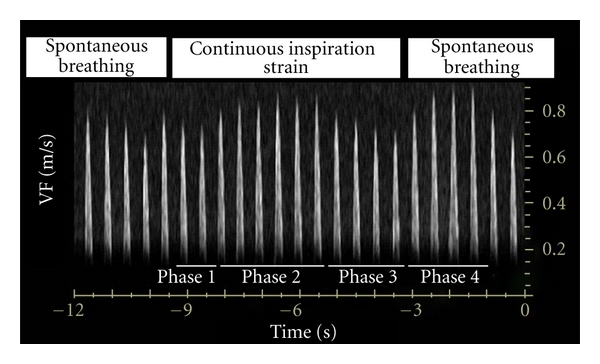
Velocity peak of femoral artery flow (VF) during a deep inspiration maneuver (DIM). Phase 1, 2, 3, and 4 of the DIM.

VF time course was assessed during DIM in 6 healthy volunteers, and mean clinical characteristics are summarized in Table 1. During inspiration strain, VF immediately decreased (phase 1), then increased (phase 2), and eventually again decreased (phase 3). During slow passive exhalation immediately following inspiration strain, VF increased (phase 4). After quiet SB was resumed, VF returned to baseline level within 30 s. The maximum value of DIM-induced VF was recorded during phase 2 or 4 (Figure 1). VF Values during SB and DIM are reported in Table 2. Intraobserver and interobserver variabilities for ΔVFdim were, respectively, 4.7% ± 3.4% and 7.1% ± 6.5%.

### 3.2. Patients

Among 250 consecutive patients hospitalized during the study, 30 (5.8%) were evaluated for inclusion in the study. Among them, 4 (13.3%) were not included because of accessory muscles use (*n* = 3), respiratory rate of ≥ 30 (*n* = 1), and/or inspiration strain below 5 s (*n* = 4). Among the 26 eligible patients, 3 (11.5%) were excluded because of transthoracic poor insonation. Thus, 23 patients (7 females and 16 males) with a mean age of 50 ± 5 years were included in the study (Table 3). Glasgow's coma score was 15/15 for all patients. Mean simplified acute physiological score II was 31 ± 12, and 2 (8.7%) patients died during hospitalization.

**Figure 2:**
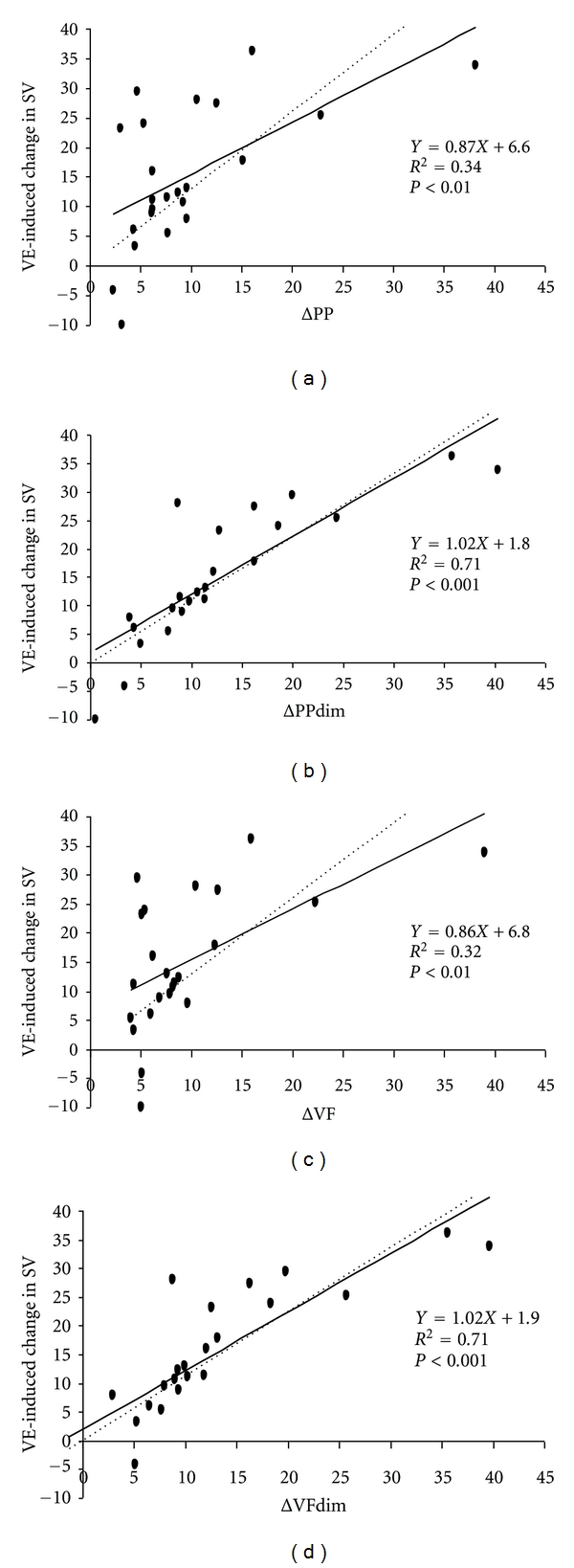
Linear correlation between respiratory change in pulse pressure (ΔPP), respiratory change in velocity peak of femoral artery flow (ΔVF), deep inspiration maneuver-induced change in pulse pressure (ΔPPdim), deep inspiration maneuver-induced change in velocity peak of femoral artery flow (ΔVFdim)- and volume expansion- (VE-) induced change in stroke volume (SV).

For the group as a whole, SV was significantly increased by VE, from 53.2 ± 12.2 mL to 62.8 ± 14 mL (*P* < 0.0001). Ten patients (43.5%) were considered responders to VE. The general characteristics of the two groups were similar prior to VE (Table 3). Invasive arterial pressure and femoral blood flow were recorded in all patients. Intraobserver variability for ΔPPdim and ΔVFdim were, respectively, 5.9% ± 4.6% and 6.3% ± 5.8%. ΔPP, ΔPPdim, ΔVF, and ΔVFdim were higher in responders than those in nonresponders (Table 4), and each was positively correlated with a VE-induced increase in SV (Figure 2). Moreover, VE-induced changes in SV were negatively correlated with VE-induced changes in ΔPP (*R* ^2^ = 0.23; *P* = 0.02), VE-induced changes in ΔPPdim (*R* ^2^ = 0.55; *P* < 0.01), VE-induced changes in ΔVF (*R* ^2^ = 0.24; *P* = 0.02), and VE-induced changes in ΔVFdim (*R* ^2^ = 0.56; *P* < 0.01).

**Figure 3:**
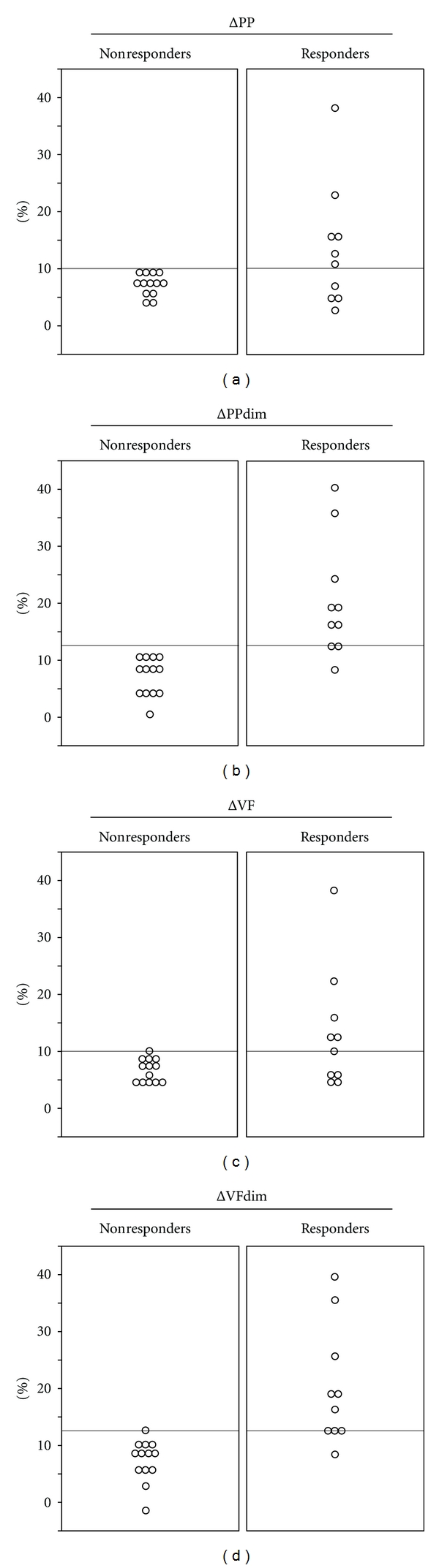
Individual baseline values for each indicator, respiratory change in pulse pressure (ΔPP), respiratory change in velocity peak of femoral artery flow (ΔVF), deep inspiration maneuver-induced change in pulse pressure (ΔPPdim), and deep inspiration maneuver-induced change in velocity peak of femoral artery flow (ΔVFdim) in patients with volume expansion-induced changes in SV ≥ 15% (responders) and <15% (nonresponders).

 AUROC ± SE for ΔPPdim (0.95 ± 0.05) and ΔVFdim (0.95 ± 0.05) were higher than AUROC ± SE for ΔPP (0.71 ± 0.12) and ΔVF (0.74 ± 0.11); *P* < 0.05. ΔPPdim and ΔVFdim of ≥12% predicted fluid responsiveness with a sensitivity of 90% and specificity of 100% (Table 5, Figure 3). No adverse effect of DIM was reported.

## 4. Discussion

The main finding of this study was that ΔVFdim and ΔPPdim enable safe and accurate bedside prediction of preload responsiveness in SB patients without ventilatory support with sepsis or acute pancreatitis. ΔVFdim and ΔPPdim of ≥12% were predictive of a positive hemodynamic response to VE induced by rapid fluid infusion. Furthermore, we demonstrated that ΔVFdim and ΔPPdim are more accurate makers of fluid responsiveness than ΔVF or ΔPP. The search for predictive factors of fluid responsiveness in SB patients was justified, since fluid responsiveness occurred in only 43.5% of patients. Thus, as previously described in SB patients, VE does not consistently improve hemodynamics [4–8].

In mechanically ventilated patients, positive pressure inspiration induces cyclic increases in right atrial pressure, causing, in turn, inverse changes in venous return, right ventricular preload and ejection, and ultimately left ventricular preload. In preload-dependent patients, these cyclic changes in ventricular filling induce cyclic changes in SV, PP, and arterial blood flow, enabling prediction of a positive response to VE [19]. In SB patients without mechanical ventilatory support, negative pressure inspiration induces cyclic decreases in right atrial pressure, causing cyclic increases in venous return, right ventricular preload and ejection, and ultimately left ventricular preload. Although SB and mechanical ventilation have inversed physiological effects on cardiac preload, respiratory changes in SV or surrogates are correlated with VE-induced changes in SV [4]. As previously described [4], the sensitivity of ΔVF and ΔPP in our study was lower than that in mechanically ventilated patients [19, 22]. Nevertheless, the predictive value of ΔPP in mechanically ventilated patients can be altered when tidal volume is low [14]. Since low tidal volume may attenuate ΔVF and ΔPP values in SB patients, we hypothesized that DIM may sensitize these indices for predicting fluid responsiveness. Our results confirm that a transient increase in tidal volume due to a standardized DIM can increase ΔVF and ΔPP sensitivity for predicting responders to VE. The main strength of the specific DIM performed in this study is that it does not necessitate specific material such as a certain type of bed [9], spirometry transducers [8], or inspiratory threshold devices [23]. The main weakness of the maneuver is the lack of respiratory parameter measurements to control whether the inspiration strain is sufficient to increase venous return to the heart. Attention should be directed to the specific population selected in our study. Indeed, ΔVFdim and ΔPPdim predicted fluid responsiveness with high sensitivity provided patients were able to understand and perform an inspiratory strain of >5 s. This prerequisite may have enabled selection of patients with appropriate inspiratory capacity, permitting accurate DIM and thus accurate fluid responsiveness prediction. However, this hypothesis should be confirmed in further studies before ΔVFdim and ΔPPdim can be routinely used at the bedside to predict fluid responsiveness in SB patients.

Continuous inspiration strain leads to a significant increase in caval blood flow [24], thus increasing cardiac preload. Although the DIM performed in this study may increase cardiac preload, hemodynamic effects are more complex and DIM-induced changes in left ventricular SV are not entirely driven by preload responsiveness of the heart. As described previously, inspiration during SB not only decreases intrathoracic pressure but also increases intraabdominal pressure and lung volume. Combined effects of the three physiologic phenomena lead to an increase not only in right ventricular preload but also in right and left ventricular afterloads [25, 26]. Therefore, DIM-induced left ventricular SV changes over time are the integrative consequence of DIM-induced changes in preload and afterload changes. First, the inspiration strain-induced increase in left ventricular afterload leads to an immediate decrease in left ventricular SV, PP and VF (phase 1 of the DIM) [26–28]. Second, if the heart is preload-responsive despite increases in right and left ventricular afterload, the increase in right ventricular preload results in an increase in right ventricular SV and, 2 or 3 heartbeats later due to pulmonary transit time of blood, in an increase in left ventricular SV, PP, and VF (phase 2 of the DIM) [11–13]. Third, the inspiration strain-induced increase in right and left ventricular afterloads overwhelms preload-dependent effects, leading to a decrease in left ventricular SV, PP, and VF (phase 3 of the DIM). As previously described during deep inspiration, global equilibrium between increased venous return and increased cardiac afterload leads to an increase in intrathoracic blood volume [27, 28] and, thus, cardiac preload. Therefore, if the heart is preload-responsive, passive exhalation immediately following deep inspiration leads to an increase in left ventricular SV, PP, and VF (phase 4 of the DIM). Thus, DIM-induced increases in PP and VF during phase 2 or 4 may correlate with cardiac preload responsiveness if their relationships with left ventricular SV are not significantly altered [7]. The high sensitivity and specificity values of ΔVFdim and ΔPPdim for predicting fluid responsiveness suggest that the relationship between left ventricular SV, PP, and VF may not be significantly altered during DIM. However, it must be underlined that no patient had abdominal compartment syndrome in this study. As intraabdominal pressure may alter hemodynamic effects of DIM, these results should not be extended to patients with suspected or confirmed abdominal compartment syndrome.

Although specificity of ΔVFdim and ΔPPdim was highly efficient at detecting VE responders, false positives may occur. As previously described, a high ΔPP baseline value could reflect either preload dependence or right ventricular dysfunction [29, 30]. Indeed, in case of obstructive lung disease and/or acute right ventricular dysfunction, an inspiratory decrease in left-ventricular diastolic compliance results in an exaggeration of the normal inspiratory decrease in PP referred to as pulsus paradoxus [31]. Therefore, ΔVFdim, and ΔPPdim might be high despite the absence of preload responsiveness and may expose patients to ineffective or deleterious fluid loadings. Evaluation of right ventricular function may help to predict false positives of ΔVFdim and ΔPPdim. Unfortunately, the study population comprised few or no patients with chronic obstructive pulmonary disease or reduced right ventricular function. Consequently, further studies are needed to determine reliability of ΔVFdim and ΔPPdim in a larger population comprising patients with obstructive lung disease and acute right ventricular dysfunction.

Eventually, it must be underlined that arrhythmia leads to misinterpretation of respiratory changes in arterial blood flow parameters, and; thus, these results cannot be extended to patients without regular cardiac rhythm.

In summary, our findings suggest that in a restricted population of SB patients with severe sepsis or acute pancreatitis, ΔVFdim and ΔPPdim are accurate indices of fluid responsiveness. Analysis of ΔPPdim or ΔVFdim is easy to perform in patients who have an indwelling arterial catheter or when echographic equipment is available. However, false negatives and false positives may occur in different clinical conditions. These results should be confirmed in a larger population before validating their use in current practice.

## 5. Acknowledgments

The authors thank the Collège National des Enseignants de Réanimation and the Société de Réanimation de Langue Française for supporting the early phase of this work and, thus, encouraging achievement of the study.

## Figures and Tables

**Table 1:** Main characteristics of healthy volunteers.

	*n* = 6
Age, years	35 ± 3
Sex ratio, M/F	4/2
Body mass index	23.6 ± 3.2
Body surface area, m²	1.83 ± 0.18
HR, beats/min	72 ± 9
RR, cycles/min	17 ± 5
MAP, mm Hg	85 ± 11
PP, mm Hg	44 ± 12
SAP, mm Hg	114 ± 17
DAP, mm Hg	70 ± 8
SVi, mL/m²	33 ± 7
VF, cm/s	74.2 ± 9.8

HR, heart rate; RR, respiratory rate; MAP, mean arterial pressure; PP, pulse pressure; SAP, systolic arterial pressure; DAP, diastolic arterial pressure; SVi, stroke volume index; VF, velocity peak of femoral artery flow. Values are expressed as mean ± SD.

**Table 2:** Velocity peak of femoral artery flow during quiet spontaneous breathing and deep inspiration maneuver in healthy volunteers.

*N*	VF during quiet spontaneous		VF during deep inspiration				ΔVF (%)	ΔVFdim (%)
	breathing (cm/s)		maneuver (cm/s)					
	Inspiration	Exhalation	Phase 1	Phase 2	Phase 3	Phase 4		
1	81.2	92.2	73.2	92.1	64	92.9	12.7	13.4
2	64	72.3	59	71.2	55.4	76.5	12.2	17.8
3	71.8	82.8	62.1	82.9	51.7	88	14.2	20.3
4	75.7	83.5	68.1	82.6	52.1	87.8	9.8	14.8
5	56.1	61.3	50	66.9	36.4	64.8	8.9	17.6
6	86.8	91.6	81.3	99.6	76.3	88.8	5.4	13.7
Mean ± SD	72.6 ± 11.2	80.6 ± 11.9	65.6 ± 11	82.6 ± 12.3	56 ± 13.4	83.1 ± 10.5	10.5 ± 3.2	16.2 ± 2.7^a^

VF, velocity peak of femoral artery flow; ΔVF, respiration-induced change in VF; ΔVFdim, deep-inspiration-maneuver-induced change in VF. ^a^ *P* < 0.05 versus ΔVF. Values are expressed as mean ± SD.

**Table 3:** Descriptive clinical data of the patients.

	Responders	Nonresponders	*P*
	*n* = 10		
Age, years	47 ± 22	53 ± 22	0.69
Sex ratio, M/F	6/4	10/3	0.65
SAPS II	31 ± 15	30 ± 10	0.82
ICU stay before inclusion, days^a^	1 [0–3]	1 [0–4]	0.90
Abdominal compartment syndrome [17]	0 (0%)	0 (0%)	1
OALL	0 (0%)	0 (0%)	1
COPD	0 (0%)	1 (8%)	1
Arterial hypertension	1 (10%)	3 (23%)	0.6
LVEF <45%	2 (20%)	1 (8%)	0.56
Indication for ICU stay (on the day of inclusion)			
Sepsis	9 (90%)	11 (85%)	1
Pulmonary infections	9 (90%)	7 (54%)	0.77
Urinary tract infections	0 (0%)	1 (8%)	0.57
Abdominal infections	0 (0%)	1 (8%)	0.57
Other infections	0 (0%)	2 (15%)	0.31
Nosocomial infections	2 (20%)	6 (46%)	0.20
Acute pancreatitis	1 (10%)	2 (8%)	1
Clinical hemodynamic parameters			
Vasoactive drugs	0 (0%)	2 (16%)	0.49
Arterial hypotension	4 (40%)	7 (54%)	0.68
Oliguria	5 (50%)	6 (46%)	1
Tachycardia	8 (80%)	9 (69%)	0.66
Mottled skin	3 (30%)	4 (31%)	1

SAPS II, Simplified Acute Physiologic Score II; ICU, intensive care unit, OALL, obliterating arteriopathy of the lower limbs; COPD, chronic obstructive pulmonary disease; LVEF, left ventricular ejection fraction. ^a^Values expressed as median and interquartile range (25th-75th percentiles). Values are expressed as number (%) or mean ± SD.

**Table 4:** Hemodynamic parameters before and after volume expansion in responders and nonresponders.

*n* = 23	Before volume	After volume
	expansion	expansion
RR, cycles/min		
Nonresponders	23 ± 4	22 ± 4
Responders	23 ± 4	22 ± 4
HR, beats/min		
Nonresponders	97 ± 22	97 ± 23
Responders	112 ± 22	107 ± 18^b^
MAP, mm Hg		
Nonresponders	79 ± 13	83 ± 13^b^
Responders	80 ± 14	90 ± 16^b^
PP, mm Hg		
Nonresponders	66 ± 19	68 ± 20
Responders	63 ± 22	71 ± 26^b^
VF, mm Hg		
Nonresponders	82.1 ± 20.5	87.5 ± 22.9^b^
Responders	79.2 ± 27.8	94.6 ± 33.4^b^
SVi, mL/m²		
Nonresponders	28.7 ± 4.9	31.3 ± 5.4^b^
Responders	28.9 ± 10.7	39 ± 14.1^b^
ΔPP, %		
Nonresponders	6.6 ± 2.5^a^	4.5 ± 2.2^b^
Responders	13.5 ± 10.6	5.5 ± 2.5^b^
ΔPPdim, %		
Nonresponders	7.2 ± 3.5^a^	5.7 ± 2.5^a^
Responders	20.6 ± 10	10.2 ± 6.1^b^
ΔVF, %		
Nonresponders	6.6 ± 1.9^a^	5.1 ± 1.1^b^
Responders	13.4 ± 10.6	6.6 ± 3.1^b^
ΔVFdim, %		
Nonresponders	7.4 ± 3^a^	6.3 ± 1.8^a^
Responders	20.4 ± 10.1	10.9 ± 5.2^b^

RR, respiratory rate; HR, heart rate; MAP, mean arterial pressure; PP, pulse pressure; VF, velocity peak of femoral artery flow; SVi, stroke volume index; ΔPP, respiration-induced change in PP; ΔPPdim, deep inspiration maneuver-induced change in PP; ΔVF, respiration-induced change in VF; ΔVFdim, deep inspiration maneuver-induced change in VF; responders, patients with volume expansion-induced changes in stroke volume ≥ 15%. ^a^ *P* < 0.05 versus responders; ^b^ *P* < 0.05 versus before volume expansion. Values given as mean ± SD.

**Table 5:** Accuracy of hemodynamic parameters for predicting fluid responsiveness.

	Threshold value	Sensitivity	Specificity	PPV	NPV	PLR	NLR	AUROC ± SE
ΔPP	10%	60%	100%	100%	76%	∞	0.4	0.71 ± 0.12
ΔPPdim	12%	90%	100%	100%	93%	∞	0.1	0.95 ± 0.05^a, b^
ΔVF	10%	60%	100%	100%	76%	∞	0.4	0.74 ± 0.11
ΔVFdim	12%	90%	100%	100%	93%	∞	0.1	0.95 ± 0.05^a, b^

ΔPP, respiratory change in pulse pressure; ΔPPdim, deep inspiration maneuver-induced change in pulse pressure; ΔVF, respiratory change in velocity peak of femoral artery flow; ΔVFdim, deep inspiration maneuver-induced change in velocity peak of femoral artery flow ^a^ *P* < 0.05 versus ΔPP; ^b^ *P* < 0.05 versus ΔVF.
